# Microdevice for directional axodendritic connectivity between micro 3D neuronal cultures

**DOI:** 10.1038/s41378-021-00292-9

**Published:** 2021-09-01

**Authors:** Yixuan Ming, Md Joynal Abedin, Svetlana Tatic-Lucic, Yevgeny Berdichevsky

**Affiliations:** 1 Department of Electrical & Computer Engineering, Lehigh University, Bethlehem, PA USA; 2 Department of Bioengineering, Lehigh University, Bethlehem, PA USA

**Keywords:** Engineering, Nanobiotechnology

## Abstract

Neuronal cultures are widely used in neuroscience research. However, the randomness of circuits in conventional cultures prevents accurate in vitro modeling of cortical development and of the pathogenesis of neurological and psychiatric disorders. A basic feature of cortical circuits that is not captured in standard cultures of dissociated cortical cells is directional connectivity. In this work, a polydimethylsiloxane (PDMS)-based device that achieves directional connectivity between micro 3D cultures is demonstrated. The device consists of through-holes for micro three-dimensional (μ3D) clusters of cortical cells connected by microtrenches for axon and dendrite guidance. The design of the trenches relies in part on the concept of axonal edge guidance, as well as on the novel concept of specific dendrite targeting. This replicates dominant excitatory connectivity in the cortex, enables the guidance of the axon after it forms a synapse in passing (an “en passant” synapse), and ensures that directional selectivity is preserved over the lifetime of the culture. The directionality of connections was verified morphologically and functionally. Connections were dependent on glutamatergic synapses. The design of this device has the potential to serve as a building block for the reconstruction of more complex cortical circuits in vitro.

## Introduction

Neuronal cultures are a widely used tool in basic and translational neuroscience research. Their advantages include ease of experimental access and compatibility with high-throughput screening technologies. In vitro neuronal cell culture models have been used for studies into neurite outgrowth^1^, synaptic strength and scaling^2^, cell interactions^3–5^, and drug screening^6,7^, among many others. However, cultures created from dissociated cortical or hippocampal cells lack the precise connectivity that characterizes circuits in these brain regions. The randomness of cultured circuits prevents accurate in vitro modeling of the brain’s development and pathogenesis of neurological and psychiatric disorders. The ability to create precise neural circuits in a high-throughput compatible and accessible platform could have a transformative effect on investigations into the mechanisms of brain development and on drug discovery.

**Fig. 1:**
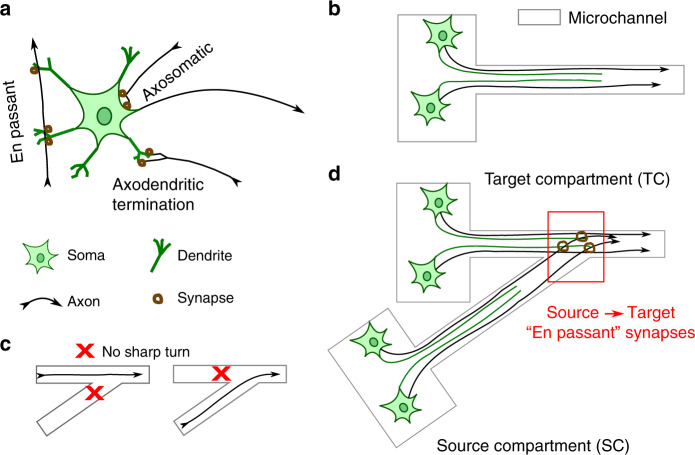
Device concept. **a** A schematic illustrating three types of synapses: axosomatic synapses, axodendritic synapses at the axon terminal and “en passant” synapses. **b** Schematic of a microchannel guiding the growth of neurites^11^. The length of the axons (black) is greater than that of dendrites (green)^23^. **c** Axons and dendrites are guided by the microchannel edge but will not follow the edge around sharp corners^12^. **d** Edge guidance and differences in axonal and dendritic lengths are exploited to create unidirectional connections from source neurons to target neurons with “en passant” synapses in this work. In the device that is schematically shown, dendrites grown by the neurons in the source compartment are too short to reach the microchannel junction. This prevents formation of TC → SC synapses. The angle of the microchannel junction is such that SC axons are edge-guided away from the neuronal soma in the TC compartment, preventing the formation of axosomatic synapses. A junction is placed close to the TC compartment so that TC dendrites can reach it. This ensures the formation of SC → TC axodendritic synapses. SC axons extend past the “en passant” synapses with TC dendrites, and can potentially be used to make further connections in more complex devices.

A basic feature of cortical circuits that is not captured in standard cultures of dissociated cortical cells is the directional connectivity. Different methods have been used to realize selective connectivity in vitro. Yamamoto et al.^8^ proposed micropatterns to build unidirectional connections between two primary neurons. This method relies on axons crossing a short cell-repellent region to form synapses with postsynaptic neurons. However, the synaptic strength was so weak that evoked presynaptic activity was unable to trigger anything more than subthreshold postsynaptic depolarizations. Peyrin et al.^9^ developed a microfluidic platform to achieve a unidirectional connection. Neurons were cultured in two compartments connected by funnel-shaped trenches. It was expected that only forward connections from the wide end of the trench to the narrow end would form. The quantification of axons in devices where one compartment was plated with cells showed promising results. However, a later study of the same device that evaluated propagation of evoked activity with both compartments plated with cells showed that only one-third of the devices had a forward-only connection^10^. A concept referred to as edge guidance was proposed: axons tend to grow along the edges of 3D guiding structures^11^ and escape it if the direction of edges changes dramatically^12^ (Fig. 1b, c). Based on this concept, optimized guiding structures have been developed for the realization of unidirectional connectivity^13–16^.

Most of the earlier work on achieving directional connectivity in vitro did not differentiate between neural soma and dendrites as axonal targets. Both were present in compartments of devices that mainly focused on controlling the direction of axon growth. Axon guidance would cease once the axon reached the somatodendritic compartment. In the cortex, most synapses are made by axons as they pass a dendrite (en passant synapses, Fig. 1a), with the axon continuing past the synapse rather than terminating^17^. This enables a single axon to make synapses with multiple dendrites belonging to different neurons. This functionality may be important for the reconstruction of neural circuits in vitro, but it was not exploited in previously reported devices. To replicate cortex-like en passant synapses, we developed a device that guides axons specifically to dendrites of target neurons (Fig. 1d). We found that continuing axon guidance past the dendrites was critical for maintaining the long-term directionality of connectivity.

Precise control over neuronal processes was enabled by the technique of confining neuronal soma in microcompartments (μ3D cultures) reported in our earlier work^7^. In this work, we report asymmetric PDMS devices of conceptually novel design (Fig. 1d) that achieved unidirectional connectivity between two μ3D cultures. We investigated the influence of geometrical parameters on the device performance. Connectivity was evaluated morphologically by axon tracing and functionally by quantifying the propagation of evoked activity. Finally, we studied the dependence of signal propagation on glutamatergic receptors.

## Results

### Device design and optimization

**Fig. 2:**
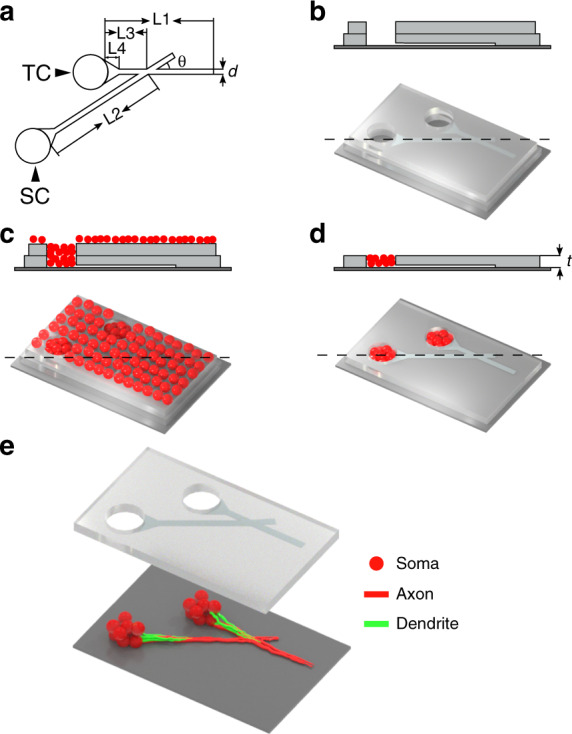
Device layout and cell plating method. **a** A schematic of the PDMS device demonstrating characteristic dimensions. **b–d** 3D schematics (bottom) and cross-sections (top) of positions indicated by dashed lines of fabrication steps and cell plating (see Materials and methods). **e** Illustration of μ3D cultures and growth of axons and dendrites. All panels are not to scale.

The device (Fig. 2) contained two through-holes serving as the target compartment (TC) and source compartment (SC) to hold µ3D neuronal cultures. Two microtrenches extended from the compartments and guided neurite growth to achieve selective TC to SC connectivity. The design of the trenches was inspired by edge guidance^12^: the neurites of neurons are prone to grow along the edges of microtrenches unless they encounter an abrupt change in the direction. The funnel-shaped end (taper) of the trenches was inspired by the diode channel structure reported previously^9^ to guide as many neurites into the trenches as possible and regulate neurite growth by reducing bouncing between the sidewalls.The characteristic dimensions of the device (Fig. 2a), including length of the trenches (*L1* and *L2*), distance between the joint of trenches and TC (*L*3), length of the taper (*L*4*)*, and width of the trenches (*d*), were optimized to maximize synaptic connections form from SC to TC and minimize connections from TC to SC, as described below.

**Fig. 3:**
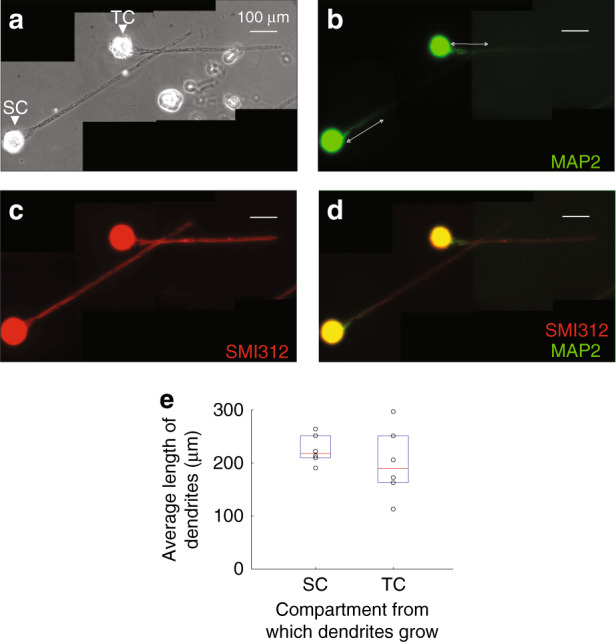
Axons and dendrites in the device. Phase contrast **a** and staining **b–d** micrographs of cultures after removal of PDMS devices. Cultures were fixed and stained on DIV 13 for MAP2 (dendritic marker) and SMI312 (axonal marker). Scale bars: 100 μm. Arrows in panel **b** denote the measured length of dendrites. **e** A boxplot of the length of dendrites growing from SC and TC. Boxes represent 1^st^ to 3^rd^ quartiles. Red bars represent medians. Whiskers represent upper and lower limits. The Wilcoxon rank sum test showed no significant difference (*n* = 6 PDMS devices from two cultures, *p* = 0.31).

First, we determined the minimum length *L*2 of the distance between SC and the trench intersection (joint). When *L*2 was too short (e.g., 200 μm), dendrites from SC reached the joint of the trenches (Supplemental Fig.S1a), potentially leading to the formation of undesired synaptic connections between axons from TC and dendrites from SC. We determined that the lengths of dendrites (MAP2^+^ processes) growing from SC and TC were 218 μm [210, 252] and 189 μm [163, 251], respectively (median [Q1 (1^st^ quartile), Q3 (3^rd^ quartile)], Fig. 3). The Wilcoxon rank sum test did not show significant differences in dendrite growth between the SC and TC compartments (*n* = 6 devices, *p* = 0.31). Thus, *L*2 was set to 600 μm, which was sufficient to prevent any dendrites from SC reaching the joint of the trenches. A short *L*3 could potentially lead to more desirable “en passant” synapses as more dendrites from the TC reach the trench joint. However, staining results showed that both dendrites and axons from TC turned toward SC when the joint of trenches was too close to TC (Supplemental Fig. S1b). However, for devices with longer *L*3, no obvious turning of axons was found (Supplemental Fig. S1c). The minimum length of dendrites was 113 μm, indicating that *L*3 set to 100 μm was short enough so that dendrites growing from TC could reliably reach the joint of trenches. On the other hand, *L*3 = 100 µm was sufficiently long so that axons and dendrites growing from TC would align with the direction of the channel prior to entering the joint region, as shown in the next section.

**Fig. 4:**
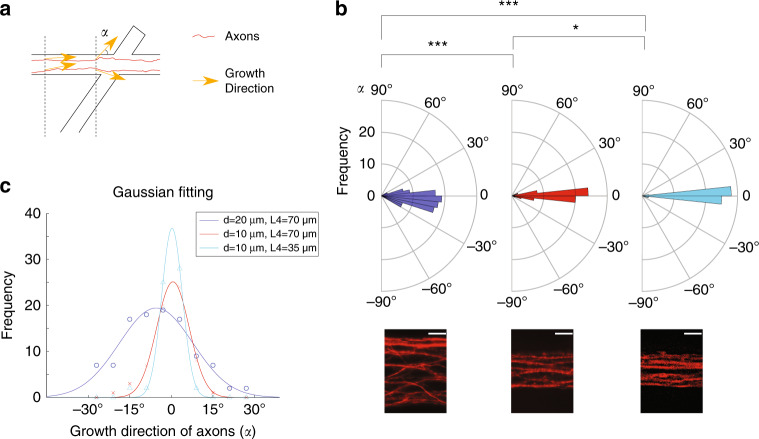
Effect of trench width *d* and taper length *L*4 on trajectories of axon growth. **a** Tangents to axons were drawn at positions indicated by dashed lines just before axon entry to the trench joint. Angle *α* between the axon tangent and trench axis represents the growth direction. **b** Polar histograms of axonal growth directions in the three geometries. Angles ranging from −90° to 90° were separated into 30 bins with an equal bin width of 6°. Color codes are the same as in panel (**c**). K–S tests were used to evaluate statistical significance. *p < 0.05, ***p < 0.001. n = 5 devices and 108 axons for *d* = 20 μm, *L*4 = 70 μm (blue), *n* = 5 devices and 66 axons for *d* = 10 μm, *L*4 = 70 μm (red), and *n* = 6 devices and 59 axons for *d* = 10 μm, *L*4 = 35 μm (cyan). At the bottom are representative fluorescent micrographs for axons in each geometry. Scale bar: 5 μm. **c** Histograms were fitted with a Gaussian function $f = ae^{ - \left( {\frac{{\alpha - b}}{c}} \right)^2}$, where *f* is the frequency and *α* is the growth direction. Fitting was performed using the MATLAB curve fitting toolbox with the nonlinear least squares method. *a* = 19.44, *b* = −5.48, *c* = 18.79, *R*^2^ = 0.94 for the blue group, *a* = 25.14, *b* = 0.56, *c* = 8.27, *R*^2^ = 0.98 for the red group, *a* = 36.78, *b* = 0.25, *c* = 5.25, *R*^2^ = 0.99 for the cyan group. Circles, crosses, and triangles are experimentally measured frequencies for each bin in the histogram (same data as in (**b**).

Axons should grow parallel to the walls of the trench before entering the joint to achieve the desirable result shown in Fig. 1c. We found that the trajectory of axon growth was related to the length of taper *L*4 (with *L*3 set to 100 µm) and the width *d* of the trenches. To quantify the directionality of axons in devices with different *L*4 and *d*, tangents to axons were drawn at positions indicated by two dashed lines in Fig. 4a. The growth direction was measured as the angle *α* between the axon tangent and the axis of the trench. Wide trenches with a long taper (*d* = 20 μm and *L*4 = 70 μm) showed a wider spread of growth directions (standard deviation stdev of *α* = 13.3°, Fig. 4b,c), indicating relatively poor guidance of axons just before their entry to the trench joint. Narrowing the trench width to *d* = 10 μm significantly decreased the spread of growth directions to stdev of *α* = 5.9° (*p* < 0.001, K–S test). Decreasing the taper to *L*4 = 35 μm further decreased the spread of growth directions to stdev of *α* = 3.7° (*p* < 0.05, K–S test).

**Fig. 5:**
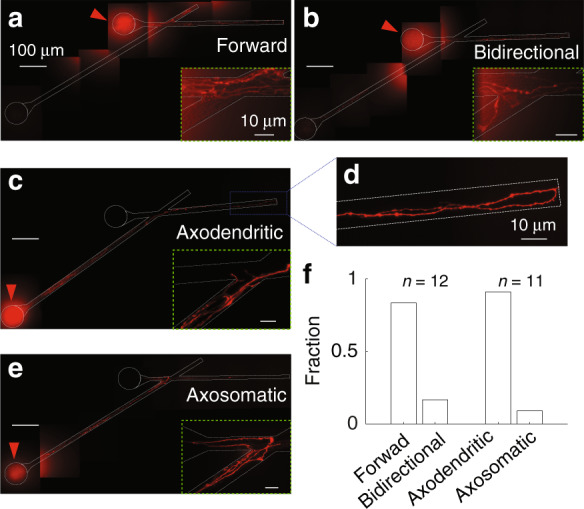
DiI axon and dendrite tracing. Cultures were fixed on DIV12. DiI crystals applied at TC showed axons not turning (**a** forward connection) and turning (**b** bidirectional connection) at the joint of the trenches. DiI crystals applied at SC showed axons not turning (**c** axodendritic connection) and turning (**e**, (partial) axosomatic connection) at the joint. Figures were generated by stitching fields of view taken with a 60X objective. White dashed lines show the outlines of microtrenches and through-holes. Inserts inside green boxes show higher-magnification views of the joints. Red arrows indicate the compartments to which DiI was applied. **d** Zoomed-in view of an axon reaching the end of the trench and making a U-turn. Scale bars in panels (a–c) and (e): 100 μm and in panel (d) and insets: 10 μm. **f** Fractions of devices with forward vs. bidirectional and axodendritic vs. axosomatic axon growth.

To confirm that the optimum geometry resulted in the desired axonal guidance, as indicated in Fig. 1c, we utilized lipophilic DiI crystals to label neurites extending from one of the compartments. DiI crystals were applied on DIV12 on cultures in geometry 30° *S* (*θ* = 30°, *d* = 10 µm, *L*4 = 35 μm, short taper is indicated by *S*). When incorporated in the neuronal membrane, DiI became strongly fluorescent and started to diffuse laterally through the membrane. DiI was applied to only one compartment of a device. When DiI was applied to the TC and no axon turned at the joint toward the SC, the device was classified as achieving “forward connection” (Fig. 5a). If axons turned toward the SC, the device was classified as “bidirectionally connected” (Fig. 5b). When DiI was applied to the SC, the device was classified as containing an “axodendritic connection” if no axon turned (Fig. 5c) toward the soma in the TC or “axosomatic connection” if axons turned toward the soma in the TC (Fig. 5e). The fractions of forward and axodendritic connections were 0.83 (*n* = 12 devices) and 0.91 (*n* = 11 devices), respectively. Surprisingly, we found in one device that axons growing from SC had already reached the end of the trench and made a U-turn (Fig. 5d), which was equivalent to more than 900 μm outgrowth of the axons on DIV 12. Axons from TC that made a U-turn at the end of the trench may extend toward SC and form connections from TC to SC, which would impact the performance of the devices. This may be the reason for the loss of unidirectionality at later time points in the functional testing of this design described below.

**Fig. 6:**
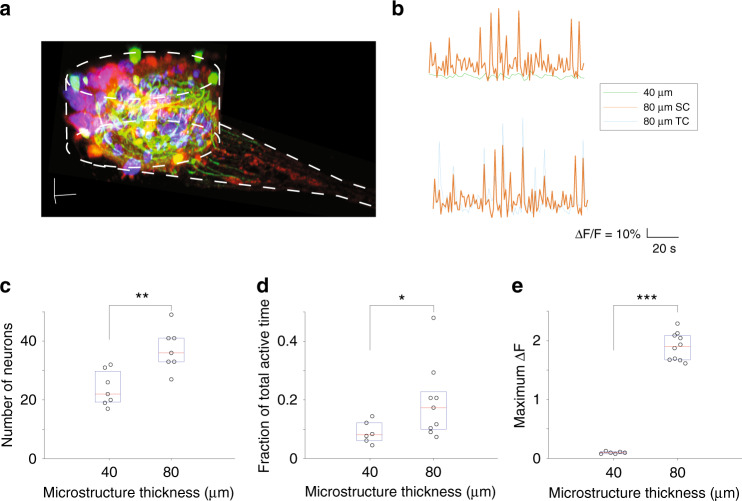
Activity in μ3D cultures in devices of different thickness. **a** 3D reconstruction of a representative μ3D culture. Dashed white lines indicate the PDMS well and the trench (microchannel). The culture was fixed and stained on DIV14 for MAP2 (green, soma and dendrites), SMI312 (red, soma and axons) and DAPI (blue, nuclei). Scale bars in x, y, and z directions: 10 μm. **b** Representative fluorescence changes caused by spontaneous activities of μ3D cultures in 40-μm-thick devices (green), SC (orange), and the corresponding TC (cyan) in an 80-μm-thick device. Number of neurons (**c**), fraction of total active time (**d**), and maximum fluorescence changes (**e**) for devices with different thicknesses. In panels c–e, each circle represents a μ3D culture. Boxes represent first to third quartiles. Red bars represent medians. The Wilcoxon rank sum test was applied to evaluate statistical significance. *p < 0.05, **p < 0.01, ***p < 0.001.

Morphological evaluation alone is not sufficient to validate directional connectivity. Functional validation requires evoking activity in one compartment and measuring propagation by detecting the activity in the other compartment. We found that the number of neurons in each compartment strongly depended on the depth of the well (thickness *t* of the PDMS shown in cross-section in Fig. 2d). For thin (40 μm) and thick (80 μm) devices, all parameters were identical except the thickness *t*. Cell seeding densities were sufficient to fill up the compartments so that the number of neurons was limited only by the thickness. In 40-μm-thick devices, the number of neurons was 22 [19.5, 28.5] (median [Q1, Q3]), which was significantly lower than that in 80-μm-thick devices (36 [33, 41], *p* < 0.01, Wilcoxon rank sum test, Fig. 6c). The μ3D cultures in thinner devices had significantly lower activity levels, including total active time (*p* < 0.05) and maximum fluorescence changes (*p* < 0.001, Wilcoxon rank sum test, Fig. 6d, e). Cultures in 80-μm-thick devices were characterized by spontaneous occurrence of population activities with high fluorescence changes and long durations^7^. These population activities in SCs successfully triggered responses in TCs (Fig. 6b), indicating the presence of transcompartment synapses. Based on these findings, we carried out functional validation in devices with *t* = 80 μm thickness.

Before performing functional validation of the diode devices, we examined the evoked responses at different stimulation intensities (Supplemental Fig. S2). We found that minimum optical and electrical stimulation intensities to evoke activities were higher on DIV 12 than on older DIVs (Supplemental Fig. S2a,b). This was likely due to synaptic immaturity and low expression levels of ChR2 on DIV12. Overall activity levels of evoked responses increased as stimulation increased (Supplemental Fig. S2c,d). Stimulation levels 1, 2, and 3 were defined as stimulation intensities that barely evoked responses (responded in < 5 out of 10 epochs and ∆ *F*⁄*F* < 0.2) and intermediate and maximum stimulation intensities, respectively. For both optical and electrical stimulations, the maximum fluorescence changes of evoked activities at level 1 were significantly lower than those at levels 2 and 3. The medians of fluorescence changes increased gradually and were significantly different at all stimulation levels. Our interpretation is that evoked activities depended on both stimulation intensity and neuronal network state within the μ3D culture. Intermediate stimulation was able to trigger network synchronizations labeled with large fluorescent changes in some epochs. When stimulation increased further, network synchronizations were triggered in all 10 epochs.

**Fig. 7:**
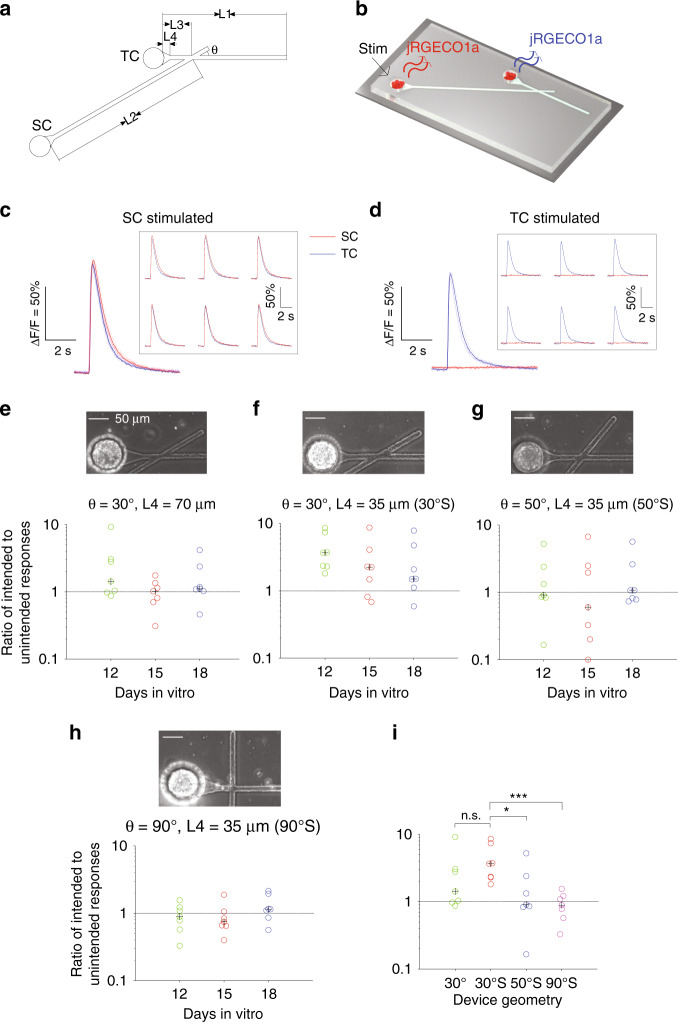
Functional evaluation of the connection directionality. **a** A schematic of the PDMS device. **b** Experimental setup when neurons in SC were stimulated. **c** Representative fluorescence changes when neurons in SC and **d** TC were stimulated. Thin lines and traces inside the inset boxes are individual epochs of the same trial. Thick lines are the averages of the six epochs (see Materials and methods). Scale bars are ∆*F*/*F* = 50% and 2 s. **e–h** Ratios of intended responses to unintended responses of different geometries on DIV 12, 15, and 18. **i** Comparison of different device geometries on DIV 12. In panel (**e–i**), each circle denotes a PDMS device (*n* = 7 for each geometry and DIV), and the black cross represents the median. Above the dashed line (ratio > 1) are selective connections from neurons in SC to TC. In panel (**i**), S in labels of x axis stands for *L*4 = 35 μm. Wilcoxon rank sum tests were used to evaluate statistical significance. *p < 0.05, ***p < 0.001 and n.s.: no significance. *n* = 7 PDMS device for each geometry.

Testing functional connections by studying the propagation of evoked responses is essential to evaluate the performance of the device. In previous studies, staining of cultures showed growth of axons predominantly in the forward direction, whereas functional tests showed evoked responses propagated in both directions^9,10^. A pair of bipolar electrodes was used to test the functional connection of cultures in the devices (Fig. 7). The performance of each geometry on different DIVs was evaluated by the ratio of intended responses to unintended responses (see Materials and methods). When the incident angle *θ*= 30°, the ratios on DIV 12 were (median [Q1, Q3]) 1.42 [1.01, 2.93] for geometry 30° (*θ* = 30°, *L*4 = 70 μm) and 3.68 [2.33, 5.56] for geometry 30° S (*θ* = 30°, *L*4 = 35 μm, S indicates a shorter taper), indicating predominantly forward connections. The performance of both geometries worsened on DIV 15 (1.02 [0.76, 1.24] and 2.24 [1.15, 3.18]) and 18 (1.11 [1.06, 1.78] and 1.51 [1.30, 3.41]), and our explanation is that more axons from the TC reached the end of trenches at later time points, made U-turns, and formed undesired functional connections with neurons in the SC. For geometry 50° S (*θ* = 50°, *L*4 = 35 μm), the ratios on DIV 12, 15, and 18 were (median [Q1, Q3]) 0.91 [0.85, 1.86], 0.60 [0.26, 2.22], and 1.07 [0.80, 1.84], respectively. For geometry 90°S (*θ* = 90°, *L*4 = 35 μm), the ratios on DIV 12, 15, and 18 were (median [Q1, Q3]) 0.89 [0.68, 1.15], 0.75 [0.66, 0.95], and 1.14 [0.98, 1.56], which showed almost equivalent connections in both forward and backward directions. We then compared the performances of different geometries on DIV 12 (Fig. 7i). Geometry 30°S was significantly better than 50° S and 90° S, which was consistent with previous studies^12,13^. The performance of 30°S was better than 30°, but no significance was found (*n* = 7 devices, Wilcoxon rank sum tests). The worse performances of 30° and 30° S on older DIVs indicated that better design of axon termination was required.

### Device finalization and evaluation

**Fig. 8:**
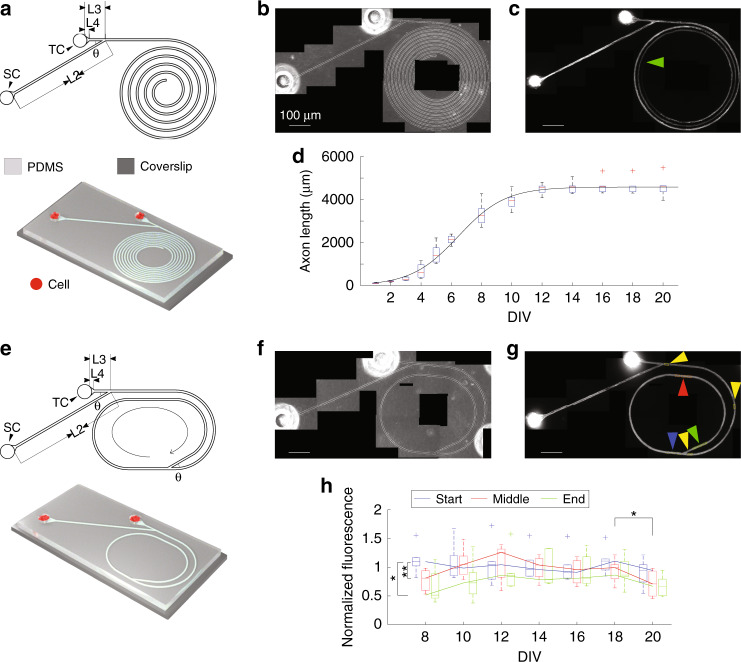
Finalized PDMS devices. (**a**) A schematic of the spiral device and (**e**) the infinite loop device. *L*2 = 600 μm, *L*3 = 100 μm, *L*4 = 35 μm and *θ* = 30°. The length of the trench in the spiral is 10 mm (measured from the joint to the end of the spiral). Phase contrast micrographs (**b, f**) and the corresponding fluorescent micrographs (**c, g**) showing the expression of ChR2-GFP on DIV 14. The green arrow in panel (**c**) indicates the terminus of axons. **d** A boxplot of lengths of axons on different days of culture. Boxes represent 1^st^ to 3^rd^ quartiles. Red bars represent medians. Whiskers represent upper and lower adjacent. Red crosses represent outliers. (*n* = 6 spiral devices). The black line denotes a curve generated by fitting medians to the sigmoid function *y* = *c*/(1+10^a(DIV-b)^) using nonlinear least squares in MATLAB. *a* = −0.646, *b* = 6.46, *c* = 4583, *R*^2^ = 0.996 and *p* = 1.06 × 10^–14^. **h** Axon growth in an infinite loop estimated by relative fluorescence intensity. Fluorescence intensities were measured from the corresponding colored boxes (indicated by colored arrows) in panel (**g**) and normalized by the references (yellow boxes in panel (**g**)). Solid lines connect medians on different DIVs. Boxes represent 1^st^ to 3^rd^ quartiles. Bars inside the boxes represent medians. Whiskers represent upper and lower adjacent. Crosses represent outliers. Wilcoxon signed-rank tests were used to measure the statistical significance of the same position between different DIVs (horizontal bracket). Wilcoxon rank sum tests were applied to calculate statistical significance between different positions on the same DIV (vertical brackets). *p < 0.05, **p < 0.01. *n* = 6 infinite loop devices.

To improve the performance of our last design, we proposed two new designs: “spiral” (Fig. 8a) and “infinite loop” (Fig. 8e). From the results of the last design of devices, we finalized our characteristic dimensions as follows: *L*2 = 600 μm, *L*3 = 100 μm, *L*4= 35 μm, and *θ* = 30°. For the spiral, we simply extended the total length of the trench to 10^4^ μm. In the infinite loop, we added one more bifurcation to the trench, expecting axons to become trapped inside the loop (as the curled arrow indicates in Fig. 8e).

We measured axon growth from cultures in spiral and infinite loops (Fig. 8). Axon length was measured using phase contrast graphs from the edge of the TC. Starting at DIV 8, when ChR2 started to be expressed, fluorescent graphs were used to confirm the measurements of axon length. Axon growth accelerated and reached the maximum rate at approximately DIV 6 when the axon length was (median [Q1, Q3]) 2154 [1956, 2320] μm. The length of axons reached a peak of 4589 [4326, 4741] μm on DIV 14. For cultures in infinite loops, axons fully occupied trenches as early as DIV 6. Since then, axons and growth cones became undistinguishable. Thus, we estimated axon growth with relative fluorescence intensities. Three yellow boxes outside the loop were selected as references. Fluorescence intensities from the start (blue), middle (red) and end (green) of the loop were measured and normalized to the references. The travel distances to reach the start, middle and end of the loop were 970, 1635, and 2300 μm, respectively. The relative fluorescence intensity at the start on DIV 8 was significantly higher than that at the middle (*p* < 0.01) and end (*p* < 0.05, n = 7 devices for all three positions, Wilcoxon rank sum test). Intensity at the start stayed high from DIV 8 to 18. The fluorescence intensity in the middle increased and reached a peak at 12 DIV. The fluorescence intensity at the end increased and reached a peak at DIV 18. There observations indicated that axons had finished the 1^st^ loop and started the 2^nd^ loop on DIV 8, and they reached the middle on DIV 10–12 and the end on DIV 14–18 for the 2^nd^ time. Significant drops in fluorescence intensities for all 3 positions on DIV 20 compared to DIV 18 (*p* < 0.05, *n* = 7 devices, Wilcoxon signed-rank test) were likely related to degeneration of axons (as expected of axon branches that did not form synapses).

**Fig. 9:**
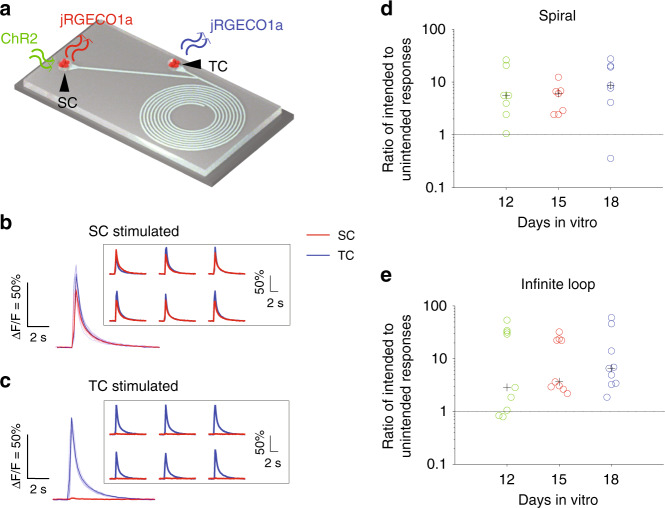
Functional evaluation of the spiral and infinite loop devices. **a** Experimental setup for the spiral and infinite loop PDMS devices when neurons in SC were stimulated. Cultures expressed jRGECO1a and ChR2. Blue light pulses were delivered to either SC or TC via a patterned illuminator, and fluorescence changes in jRGECO1a were recorded (Materials and Methods). **b** Representative fluorescence changes when neurons in SC, and **c** TC were stimulated. Thin lines and traces in insets are individual epochs of the same trial. Thick lines are the averages of the six epochs (see Materials and methods). Scale bars are ∆F/F = 50% and 2 s. Ratio of intended responses to unintended responses of spiral (**d**) and infinite loop (**e**) PDMS devices. Each circle denotes a PDMS device (*n* = 7 for spiral and 9 for infinite loop), and the black cross represents the median. Above the dashed line (ratio > 1) are the selective connections from neurons in SC to TC.

Next, we tested functional connections using cultures expressing both ChR2 and jRGECO1a (Fig. 9). The ratios of intended responses to unintended responses for spirals on DIV 12, 15, and 18 were 5.55 [3.19, 13.14], 6.06 [2.66, 6.71], and 8.69 [5.91, 19.74], respectively. Wilcoxon rank sum tests did not show significance between DIVs, *n* = 7 spiral devices. The ratios for the infinite loop on DIVs 12, 15, and 18 were 2.86 [1.06, 32.09], 3.65 [2.95, 22.53], and 6.60 [3.44, 14.07], respectively. Wilcoxon rank sum tests did not show significance between DIVs, *n* = 9 infinite loop devices. Both spiral and infinite loops showed that predominant forward connections prevailed on all DIVs, thus achieving better performance than the previous design.

The yield of the finalized devices was also evaluated (Supplemental Fig. S3). Each PDMS device array contained 16 devices. From phase contrast pictures, μ3D cultures without obvious volume shrinkage were counted as “healthy cultures” (an example of an unhealthy culture is shown in Supplemental Fig. S3b). Functional tests of spiral and infinite loop devices were performed only on “healthy cultures”. All μ3D cultures marked as “healthy cultures” within the tested devices responded to the optical stimulation. Devices were designated “connected devices” if TC activity was evoked by stimulation of SC, or vice versa. The ratio of SC → TC response vs. TC → SC response is shown in Fig. 9d,e . Fractions of “healthy cultures” and “connected devices” were 0.92 ± 0.07 (*n* = 224 μ3D cultures from seven PDMS device arrays) and 0.94 ± 0.10 (*n* = 25 tested devices from five PDMS device arrays), respectively. High yield indicated the reliability and robustness of this device design and methodology.

**Fig. 10:**
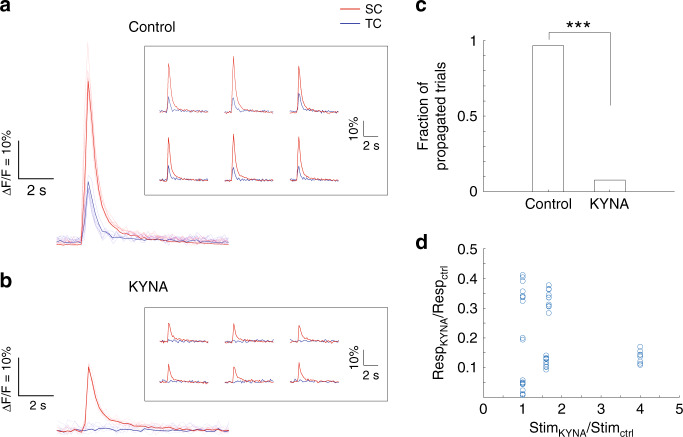
Functionality of connections between compartments depends on glutamatergic neurotransmission. Representative fluorescence changes of cultures on DIV 21 in regular recording medium (**a**), and in the presence of 3 mM KYNA (**b**). Thin lines and traces in the inset boxes are individual epochs of the same trial. Thick lines are the averages of the six epochs (see Materials and methods). Scale bars are ∆*F*/*F* = 10% and 2 s. **c** Fraction of trials in which stimulation-evoked responses in SC propagated to TC. Two proportion Z tests were used to measure statistical significance. ***p < 0.001. *n* = 30 stimulations from three spiral devices. **d** In the presence of KYNA, it was more difficult to evoke responses from cultures than under regular conditions. Resp_KYNA_ and Resp_ctrl_ represent maximum evoked fluorescence changes with KYNA and under control conditions. Stim_KYNA_ and Stim_ctrl_ represent the magnitude of stimulations to evoke responses with KYNA and in the control condition.

We then tested signal propagation in the presence of TTX and KYNA. No responses were evoked with stimulation when TTX was applied (results not shown). The presence of KYNA significantly reduced the fraction of propagation of evoked responses (Fig. 10c, *p* < 0.001, two proportion z test, *n* = 30 stimulations from three spiral devices). With KYNA, the magnitudes of evoked responses were lower with the same or higher stimulation intensities (Fig. 10d). These findings indicated that both generation and propagation of the responses were dependent on the glutamatergic synapses.

## Discussion

The design of the device presented in this work relies in part on the concept of axonal edge guidance developed earlier^12^ and on the novel concept of specific dendrite targeting. Targeting dendrites with a passing axon has two advantages: (1) it replicates the dominant connectivity of excitatory axons in the cortex, and (2) it enables guidance of the axon after it forms an “en passant” synapse. We found that the latter is critical for ensuring the long-term (up to DIV 18) directionality of connections, something that proved challenging in earlier studies^15^. In our early designs (Figs. 5 and 7), we found that the loss of guidance occurred due to axons extending to the end of the microchannel, making a U-turn, and potentially making unintended synaptic contacts. This problem was exacerbated by the surprising extent of axon growth in this work, reaching a length of 2 mm by DIV 6 in contrast to approximately 500 μm axon growth by DIV 6 reported by Forro et al.^15^. The maximum axon growth rate (occurring between DIV 4 and 6) in our device was ~ 33 μm/h, double the maximum axon growth velocity of ~ 15 μm/h reported by Gladkov et al.^14^. These differences may be due to the use of hippocampal neurons in earlier work, whereas we used cortical neurons. On the other hand, earlier reports used embryonic neurons that could be expected to grow axons more rapidly than the postnatal neurons used by us. Another reason for the high axon growth rate and maximum extension may be due to the improved viability of neurons in μ3D cultures compared to 2D cultures, evidenced by more developing cortex-like spontaneous activity in these cultures^7^. To the best of our knowledge, this is the first work to build directional connectivity between μ3D cultures.

To cope with long axons, we developed a final version of our design that incorporated two concepts for postsynaptic axon guidance: spiral and infinite-loop. The spiral device demonstrated that axons could extend for millimeters past the synapse formation zone. This may potentially be used in future work to daisy-chain devices into more complex neural circuits, with each axon making synapses with dendrites extended by multiple TC compartments. The infinite-loop device demonstrates axon termination in case daisy-chaining multiple devices are not required. In this situation, it may be important to minimize the surface area taken up by the axon termination component, especially if multiple devices are used in an array format for high-throughput experiments. It may be possible to further shrink the size of the loop by decreasing the curvature radius, although there may be a lower limit to the ability of the axon to follow the curve.

Evaluation of connectivity in compartmented neuronal cultures may produce different results if only one, rather than both compartments, is populated with cells. In the case where cells are seeded in one compartment only and axons that enter the other compartment are quantified by staining, the results may be misleading. The performance of the same structure decreased as much as six times when neurons were seeded in both compartments compared to the source compartment only^12^, indicating that axon rejection was affected by the presence of axons extending in the opposite direction. Similar findings were reported in another work where with neurons cultured in one compartment, the ratio of axons growing in the backward vs. forward direction was 3–5%^9^. However, a later evaluation of the same structure using an activity-based method showed that two-thirds of the cultures were bidirectionally connected^10^. Therefore, in this work, we carried out both morphological and activity-based evaluations of directionality in devices with both compartments populated by cells.

Activity-based evaluation was carried out by either electrical or optical stimulation. Delivery of the electrical stimulation to a specific compartment was straightforward—bipolar electrodes were placed onto the μ3D cultures under microscope control. Spatial selectivity of optical stimulation (blue light activation of ChR2 expressed in all neurons in the cultures) required the use of a patterned illuminator that delivered light to the selected compartment. Despite its higher complexity, optical stimulation has the important practical advantage of not requiring breaking the sterility of the culture for electrode placement. This allowed us to evaluate activity propagation in the same culture on different DIVs, thus decreasing the number of cultures that had to be generated. μ3D cultures fire spontaneous population bursts^7^. To guarantee that observed activities were evoked by stimulus, we used a recording medium with elevated [Mg^2+^] to suppress spontaneous bursting (see “Materials and methods”).

In finalized devices, the ratio of intended responses to unintended responses reached peaks of 8.69 (median) for “spiral” and 6.60 (median) for “infinite loop” on DIV 18 (Fig. 9d, e), which was likely due to maturation of synapses^18^. Variances of device performances on different DIVs were also reported in previous studies. Gladkov et al.^14^ evaluated the performances of asymmetric channels linking source and target compartments as a ratio of evoked bursts propagated in forward and backward directions. The best ratio was reported to average 7 on DIV 20. Degradation of performance by DIV 25 (ratio of ~ 2.5) was likely due to a small portion of axons from the target compartment escaping restrictions of the asymmetric channels and reaching the source compartment on late DIVs. Forro et al.^15^ evaluated the performance by calculating transfer entropies (directed information flow) in forward and backward directions generated from spontaneous bursts. The best performance was observed on DIV 15, and the ratio of median transfer entropy in the forward direction to that in the backward direction was approximately 8.76. Axons growing in undesired directions were observed at late DIVs, which resulted in degradation of performance on DIV 18. The rectification performance of our finalized devices therefore matches the peak results of previously reported devices without performance degradation at later time points, due to nonspecific axon growth.

In previous studies of directional connectivity, axons were guided from source neurons directly to target neurons with both soma and dendrites as targets of synapses^10,12–16^. Compared to previous studies, our devices targeted dendrites specifically (“en passant” synapses). Since the peak performance was comparable to earlier devices, this work represents a proof-of-concept that directional connectivity in vitro can be achieved by guiding axons to dendrites as opposed to soma. Our devices can potentially be adapted to target dendrites at different locations to create proximal and distal synapses to accurately model cortical development and synaptic integrations. Another advantage of our devices is that they continue to guide axons after they pass dendrites of target neurons (Fig. 1), which makes it possible to form connections from one source to multiple independent targets.

The amplitude of evoked activity, exceeding 10% Δ*F*/*F* in most cases, suggested that both electrical and optical stimulation evoked bursts rather than single action potentials (single action potential Δ*F*/*F* for jRGECO1a, the Ca^2+^ indicator used in this work, is typically < 3% for cultured cortical neurons). This is further evidenced by a lower amplitude of evoked activity, even in response to higher applied stimulation, in the presence of the glutamatergic antagonist KYNA. The effect of KYNA suggests that evoked bursting depended on glutamatergic synapses within each μ3D culture. Despite its burst-like nature, we found that evoked activity was not all-or-nothing but rather scaled with the amplitude of both electrical and optical stimulation. This allowed us to scale the evoked responses in both source and target compartments such that their amplitudes were approximately equal. This in turn ensured that quantification of propagated activity was not biased by the amplitude of evoked activity in the results reported in Figs. 7 and 9. However, such bias was unavoidable in experiments that examined propagation in the presence of KYNA (Fig. 10). Nevertheless, the nearly complete absence of activity propagation in KYNA strongly suggests that “en passant” synapses made by SC axons with TC dendrites were glutamatergic.

In conclusion, we demonstrated PDMS devices with asymmetric microtrenches based on edge guidance of axons to achieve selective connections between two μ3D neuronal cultures of < 100 μm in size. We verified unidirectional connections morphologically by DiI staining and functionally by propagation of evoked activity. We also demonstrated that responses to stimulation and signal propagation between cultures were dependent on glutamatergic synaptic activity. This is the first reported method to construct unidirectional connections between μ3D cultures. Due to the microscale size and ability to form “en passant” axodendritic synapses, the developed devices have the potential to serve as a starting point to build more complicated neuronal circuits in vitro.

## Materials and methods

### PDMS device array preparation

The detailed fabrication method is published elsewhere^7^. Briefly, a 2-μm-thick layer of SU-8 2 (Kayaku Advanced Materials Inc.) was fabricated on a 3-inch silicon wafer to define the trench layer. Next, an 80-μm-thick layer of SU-8 2050 (Kayaku Advanced Materials Inc.), which defined the compartment layer, was aligned with the first layer. Polydimethylsiloxane (PDMS) base and curing agent (Dow Corning Corp.) were mixed at a 10:1 ratio, spun at 800 RPM (revolutions per minute) and cured on the SU-8 master. Then, cured PDMS device arrays were peeled off, cut to the desired size and cleaned with ethanol. Two layers of PDMS device arrays were aligned and attached to a PDL (poly-D-lysine, Sigma-Aldrich)-coated coverslip (Fig. 2b). Finally, the device arrays were placed into a Petri dish and maintained in 2 mL of serum-free culture medium (97.5% Neurobasal-A medium (Invitrogen), 2% B27 (Gibco), 0.5 mM GlutaMAX (Gibco) and 30 μg/mL gentamicin (Gibco)) in an incubator overnight.

### Neuronal cell culture

Primary neurons were obtained from Sprague–Dawley rat pups (Charles River Laboratories) at stages P0–1 (postnatal day 0–1). All animal use protocols were approved by the Institutional Animal Care and Use Committee (IACUC) at Lehigh University and conducted in accordance with the United States Public Health Service Policy on Humane Care and Use of Laboratory Animals. Dissection and cell culture were performed per an established protocol.^19^ Brains were microdissected in Hanks’ balanced salt solution (HBSS, Gibco) on ice. Cortices were digested in papain solution (Worthington Biochemical Corp.) at room temperature for 20 min. After digestion, cortices were transferred to 5 mL of fresh culture medium. The supernatant was discarded, and the cortices were washed with fresh culture medium two more times. Next, the supernatant was replaced with 5 mL of culture medium. The cortices were triturated carefully, and the cell solution was mixed with 5 mL of Percoll solution (1.5 mL Percoll (Sigma-Aldrich), 0.5 mL 10X HBSS (Gibco), and 3 mL distilled water (Invitrogen)). Cell pellets were obtained after centrifugation and resuspended in cell plating medium (Neurobasal-A, 0.5 mM GlutaMAX, 30 μg/mL gentamicin, and 10% fetal bovine serum (FBS, Gibco)) to a density of 2.54 × 10^7^ cells/mL. PDMS device arrays were cleared of bubbles in a vacuum desiccator and washed two times with Gey’s balanced salt solution (Sigma-Aldrich). After aspirating the medium covering the device arrays, 20 μL of the cell solution was injected (Fig. 2c). After settling for 15 min, cells were supplied with 2 mL of cell plating medium. After 45 min, the cell plating medium was replaced with 2 mL of culture medium. Cells were maintained in a humidified, 37 °C, 5% CO_2_ incubator thereafter, and 1 mL of culture medium was replaced twice each week. The top layer of the PDMS device array was peeled off 24 h after cell plating (Fig. 2d).

### Viral infection

Cells were infected on day in vitro (DIV) 1 with an adeno-associated virus (AAV). To express the calcium sensor protein jRGECO1a^20^, cells were infected with pAAV.Syn.NES-jRGECO1a.WPRE. SV40 (a gift from Douglas Kim & GENIE Project (Addgene plasmid # 100854; http://n2t.net/addgene:100854; RRID:Addgene_100854)) at titer ≥ 5 × 10^9^ vg/mL. For optogenetic activation of cells, they were infected with pAAV-hSyn-hChR2(H134R)-EYFP (a gift from Karl Deisseroth (Addgene plasmid # 26973; http://n2t.net/addgene:26973; RRID:Addgene_26973)) at titer ≥ 5 × 10^9^ vg/mL.

### Immunocytochemistry (ICC)

For optimal staining of neurites, PDMS device arrays were removed carefully. Cell cultures were washed three times with phosphate buffered saline (PBS, Sigma-Aldrich), followed by fixation with 4% paraformaldehyde (PFA, Electron Microscopy Science) in PBS at room temperature for 1 h. After washing three times with PBS, 0.3% Triton X-100 (Sigma-Aldrich) in PBS was applied for 15 min for permeabilization, and 10% goat serum (Gibco) in 0.05% Triton X in PBS was applied for 1 h for blocking. Primary antibodies against MAP2 (diluted to 1:2000, BioLegend) and SMI312 (diluted to 1:500, BioLegend) were applied to the cell culture and kept at 4 °C on an orbital shaker at 55 RPM for 72 h. Secondary antibodies were added after washing and kept at 4 °C on an orbital shaker at 55 RPM for 24 h. Cell cultures were imaged on an inverted microscope (Nikon TE2000).

For DiI staining, DiI crystals (Invitrogen) were placed at the top center of the culture confined in each compartment. Cultures were kept at 4 °C for 1 week before imaging on an inverted microscope (Nikon TE2000).

### Stimulation and calcium imaging

The recording medium used during experiments with electrical stimulation contained the following (in mM): 134.8 NaCl, 2.4 KCl, 2 CaCl_2_, 4 MgCl_2_, 10 glucose, 10 HEPES and 1.2 NaH_2_PO_4_ (pH 7.4), which was adapted from a previous study.^8^ Cell cultures were allowed to recover in the incubator for 15 min after the culture medium was replaced with recording medium. Cultures were maintained in a miniature incubator (Bioscience Tools) at 37 °C. A bipolar electrode was carefully placed on top of the culture in the compartment at the shortest possible distance without touching it (Fig. 7b). Each experimental trial contained 10 identical consecutive epochs. Each epoch lasted 10 s. After a 0.5 s delay of each epoch, a biphasic square voltage with a 0.2 ms pulse width was generated by a stimulus isolator (model 2300, A–M systems) and delivered to the electrode. The magnitude of the stimulating voltage was increased until cultures responded with similar fluorescence changes in most of the epochs within the same trial. Within the same PDMS device array, fluorescence changes of cultures in the two compartments were recorded at 5 frames per second (FPS) simultaneously using a dual-deck inverted microscope (IX73, Olympus) equipped with a CCD camera (DFK 23U618, The Imaging Source).

For optical stimulation, similar setup was used. Petri dishes containing cultures in recording medium were placed in the mini incubator, which was maintained at 37 °C. The stimulation region was defined to cover the whole µ3D culture in the SC or TC compartment using PolyScan2 software (MIGHTEX). 20 ms pulses of blue light generated by an LED (X-Cite 110 LED Illumination System, Excelitas Technologies Corp.) were delivered to the stimulation region by a Polygon400 patterned illuminator (MIGHTEX) through one deck of the dual-deck inverted microscope (IX73, Olympus). Recordings of the fluorescence changes were taken via the second deck of the same microscope equipped with appropriate filters for jRGECO1a.

### Pharmacological experiments

The electrical stimulation protocol described in the previous section was used during pharmacological experiments of kynurenic acid (KYNA, Sigma-Aldrich) and tetrodotoxin (TTX, Tocris). Culture in the source compartment (SC) was stimulated, and fluorescence changes from both SC and TC (target compartment) were recorded. Next, the extracellular medium was replaced with recording medium containing 3 mM KYNA or 1 μM TTX. After 15 min of incubation, culture in the SC was stimulated with the same or higher voltage. Fluorescence changes from both SC and TC (target compartment) were recorded.

### Data analysis

Regions of interest (ROIs) were drawn to include the whole culture in each compartment. ImageJ^21^ was used to extract the mean gray value *F* of each ROI. Asymmetric least square smoothing^22^ was implemented in MATLAB to calculate the baseline *F*_0_. Fluorescence change was obtained by

$${\Delta}{{F}}/{{F}} = ({{F}} - {{F}}_0)/{{F}}_0$$

The peak evoked response was defined as the maximum fluorescence change within two frames after stimulation. The average and standard deviation of the peak evoked response of ten epochs were calculated. Epochs were discarded until the ratio of standard deviation to average was less than 0.15 or only six epochs were left. Multiple trials were carried out for SC and TC of each PDMS device. Trials that evoked similar fluorescence changes in SC and TC of the same PDMS device were chosen for final data analysis. When stimulation was delivered to culture in SC at *t*_0_, the maximum fluorescence change of culture in TC from *t*_0_ to *t*_0_ + 2 *T* (*T* was the duration of each frame) was defined as the intended response. Unintended response was defined as the maximum fluorescence change of SC from *t*_0_ to *t*_0_ + 2 *T* when culture in TC was stimulated at *t*_0_. The ratio of the intended response to the unintended response was calculated to evaluate the performance of each design of the PDMS devices.

## Supplementary information

Supplemental Materials for Microdevice for directional axo-dendritic connectivity between Micro 3D neuronal cultures

## Data Availability

The raw and processed data required to reproduce these findings are available upon request.
