# Supplementary material for: Microdevice for directional axodendritic connectivity between micro 3D neuronal cultures
Source: Microsyst Nanoeng. 2021 Sep 1;7:67. doi: 10.1038/s41378-021-00292-9 (PMC8433170; doi:10.1038/s41378-021-00292-9)
Supplement: Supplementary file 1 — Supplemental Materials for Microdevice for directional axo-dendritic connectivity between Micro 3D neuronal cultures [file 41378_2021_292_MOESM1_ESM.docx]

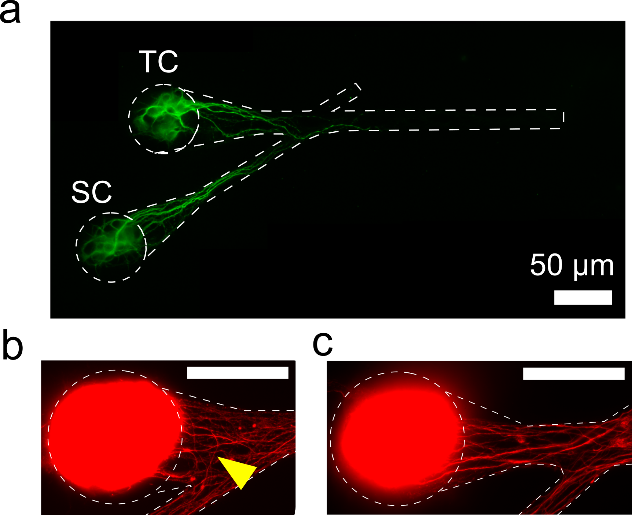


**Supplemental Figure S1**. **(a)** Fluorescent images of neurons and dendrites stained on DIV 6 with anti-MAP2. Dendrites from SC reached the joint of the trenches. **(b-c)** Fluorescent images of axons stained on DIV 6 with anti-SMI312. When the joint is too close to the TC, an axon extended from TC was found turning towards SC (b, yellow arrow). Whereas, when the joint is away from the TC, no axons were found obviously turning towards SC. Cultures were fixed and stained on DIV6. Scale bars: 50 μm.


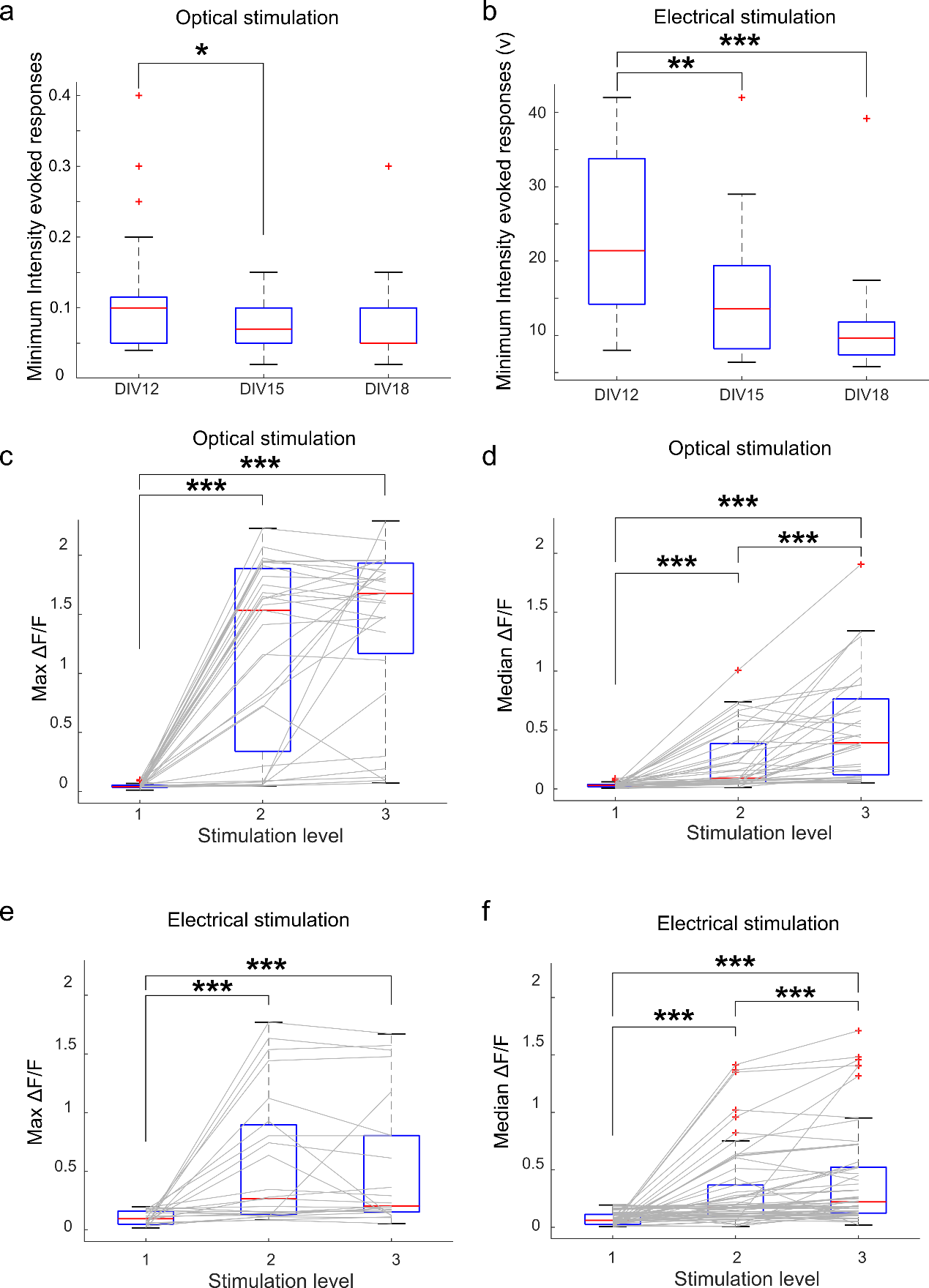


**Supplemental Figure S2**. Characteristics of evoked responses to stimulation intensities. Minimum intensities of optical **(a)** and electrical **(b)** stimulation to evoke responses on different DIVs. For optical stimulation, y axis represents the fraction of total power of the LED (160 W). Maximum **(c)** and median **(d)** of fluorescence changes at different optical stimulation levels. Maximum **(e)** and median **(f)** of fluorescence changes at different optical stimulation levels. For boxplots in all panels, boxes represent 1^st^ to 3^rd^ quantiles. Red bars represent medians. Whiskers represent upper and lower adjacent. Red crosses represent outliers. Grey lines in panels (c-f) are data from individual cultures. Statistical tests are Wilcoxon signed-rank test: (A) n = 31, (C) n = 31, (D) n = 35, (E) n = 23, (F) n = 58 cultures, and Wilcoxon rank sum test: (B) n = 24 cultures. * : p < 0.05, ** : p < 0.01 and *** : p < 0.001.


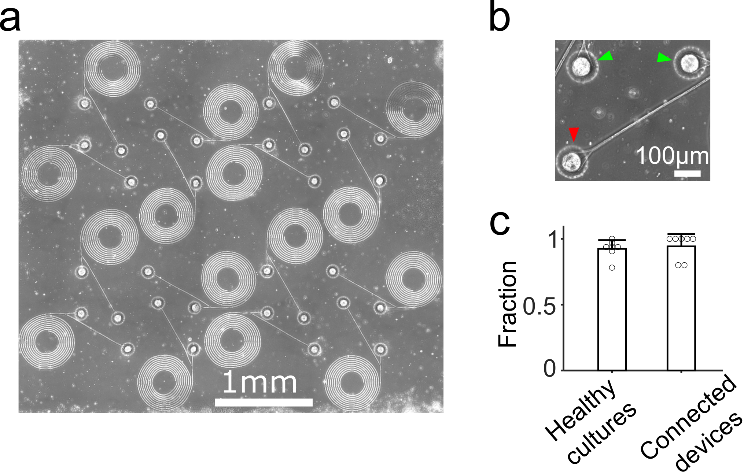


**Supplemental Figure S3. (a)** Representative phase contrast micrograph of the spiral device array. **(b)** A micrograph showing one unhealthy (red arrow) and two healthy (green arrows) μ3D cultures. “healthy cultures” were defined as μ3D cultures without obvious volume shrinkage. **(c)** Yield of the finalized devices (spiral and infinite loop). Fraction of healthy cultures is calculated by dividing number of healthy cultures by total compartments (n = 224 μ3D cultures from 7 PDMS device arrays). Functional tests of spiral and infinite loop devices were only performed on “healthy cultures”. Devices were designated as “connected devices” if TC activity was evoked by stimulation of SC, or vice versa (n = 25 tested devices from 5 PDMS device arrays). Each circle denotes a PDMS device array. Bar plots and error bars are averages and standard deviations, respectively.
